# LRIG3 Suppresses Angiogenesis by Regulating the PI3K/AKT/VEGFA Signaling Pathway in Glioma

**DOI:** 10.3389/fonc.2021.621154

**Published:** 2021-02-25

**Authors:** Chenghao Peng, Hanmin Chen, Youwei Li, Hang Yang, Peizhong Qin, Baojun Ma, Qiuhong Duan, Baofeng Wang, Feng Mao, Dongsheng Guo

**Affiliations:** ^1^ Department of Neurosurgery, Tongji Hospital, Tongji Medical College, Huazhong University of Science and Technology, Wuhan, China; ^2^ Department of Biochemistry and Molecular Biology, School of Basic Medicine, Huazhong University of Science and Technology, Wuhan, China; ^3^ Department of Neurology, Union Hospital, Tongji Medical College, Huazhong University of Science and Technology, Wuhan, China; ^4^ Department of Medical Genetics, School of Basic Medicine, Tongji Medical College, Huazhong University of Science and Technology, Wuhan, China; ^5^ Department of Neurosurgery, The Second Affiliated Hospital of Nantong University, Nantong University, Nantong, China

**Keywords:** glioma, angiogenesis, LRIG3, vascular endothelial growth factor A, PI3K/AKT

## Abstract

High levels of microvessel density (MVD) indicate poor prognosis in patients with malignant glioma. Leucine-rich repeats and immunoglobulin-like domains (LRIG) 3, a potential tumor suppressor, plays an important role in tumor progression and may serve as a biomarker in many human cancers. However, its role and underlying mechanism of action in glioma angiogenesis remain unclear. In the present study, we used loss- and gain-of-function assays to show that LRIG3 significantly suppressed glioma-induced angiogenesis, both *in vitro* and *in vivo*. Mechanistically, LRIG3 inhibited activation of the PI3K/AKT signaling pathway, downregulating vascular endothelial growth factor A (VEGFA) in glioma cells, thereby inhibiting angiogenesis. Notably, LRIG3 had a significant negative correlation with VEGFA expression in glioma tissues. Taken together, our results suggest that LRIG3 is a novel regulator of glioma angiogenesis and may be a promising option for developing anti-angiogenic therapy.

## Introduction

Currently, glioblastoma (GBM) represents the most common primary brain tumor in adults and carries a particularly poor prognosis (1). Despite the use of multimodal treatments, such as maximal surgical resection, chemotherapy, and radiotherapy, the average survival time of GBM patients, following diagnosis, is only 12 to 15 months (2, 3). This poor prognosis is mainly attributed to aberrant angiogenesis, high invasiveness, and therapeutic resistance.

Angiogenesis, a process in which new blood vessels develop from the existing vasculature, has been implicated in various physiological and pathological changes, such as tumor, development, and wound healing (4). Additionally, abnormal angiogenesis reportedly leads to proliferation, survival, and metastasis of tumors. High tumor microvessel density (MVD) has been shown to be a hallmark of GBM, with higher MVD levels associated with worse prognosis in patients (5, 6). Glioma angiogenesis entails the coordination of multiple pro- and anti-angiogenic molecules, such as vascular endothelial growth factor (VEGF) family, endostatin, and angiostatin (7). An imbalance among these factors results in abnormal angiogenesis. To date, the precise mechanisms underlying the regulation of these angiogenesis-related factors are not fully understood.

The human leucine-rich repeats and immunoglobulin-like domains (LRIG) gene family comprises LRIG1, LRIG2, and LRIG3, which encode single-pass transmembrane proteins. LRIG genes are clinically relevant prognostic indicators in several human cancers and play various roles in tumor cell proliferation, migration, invasion, apoptosis, and chemosensitivity (8, 9). LRIG3 was the last gene to be discovered in this family and is frequently downregulated in human cancers (10–12). In fact, LRIG3 acts as a tumor suppressor in GBM, where it modulates proliferation, migration, and invasion of glioma cells by targeting the EGFR and MET signaling pathways (13, 14). Moreover, LRIG3 expression is significantly higher in low-grade gliomas than in high-grade gliomas (grades III and IV), and LRIG3 upregulation suggests a better prognosis in malignant glioma patients (14). However, the actual molecular mechanisms underlying LRIG3’s role in angiogenesis are poorly understood.

In this study, we demonstrated that LRIG3 acted as an anti-angiogenic gene, inhibiting VEGFA *via* the PI3K/AKT signaling pathway. Our results suggested that LRIG3 is a potential target for the future development of therapeutic strategies against glioma angiogenesis.

## Materials and Methods

### Cell Culture and Reagents

Human glioma cell lines, U87 and U251, were purchased from the American Type Culture Collection (ATCC; Manassas, VA, USA). The Human umbilical vein endothelial cell (HUVEC) line was purchased from the Cell Bank at the Shanghai Branch of Chinese Academy of Sciences (Shanghai, China). Glioma cells were cultured in Dulbecco’s modified Eagle’s medium (DMEM; Gibco), supplemented with 10% fetal bovine serum (FBS; Gibco) and 1% penicillin/streptomycin. HUVECs were cultured in Endothelial Cell Growth Medium-2 (EBM-2) Bulletkit (Lonza, Walkersville, MD, USA). All cells were maintained in a humidified incubator at 37 °C and 5% CO_2_. PI3K inhibitor LY294002 (Cat #S1105) and MEK inhibitor PD98059 (Cat #S1177) were purchased from Selleck (Houston, TX, USA).

### Sample Collection and Study Approval

Twenty-eight glioma tissue samples were postoperatively obtained from patients at the Department of Neurosurgery, Tongji Hospital. Histological features of all specimens were confirmed by pathologists, according to the WHO criteria. A summary of patient characteristics is shown in **Supplementary Table S1**. This study was reviewed and approved by the Research Ethics Committee of Tongji Hospital, Tongji Medical College, Huazhong University of Science and Technology. Written informed consent was obtained from all participants before inclusion.

### Conditioned Medium (CM)

Glioma cells were routinely cultured for 48 h, confluent cultures washed twice with serum-free medium, then incubated in serum-free EBM-2 medium for 24 h. The supernatant was harvested after conditioning, centrifuged at 2,000*g* at 4°C for 15 min, filtered through 0.22 μm Millipore filters, then supplemented with 0.5% serum and 1 ng/ml bFGF. The contents were frozen at −20°C until required.

### Construction and Transduction of Lentiviral Vector

Lentiviral vectors were produced as previously described (15). Briefly, pLVX-DsRed-LRIG3 was constructed and transduced into U87 and U251 together with empty vector pLVX-DsRed, using Clontech’s Lenti-X™ high-titer lentiviral packaging systems (Clontech Company, USA). The generated constructs were named LRIG3 and vector control groups, respectively.

### siRNA Transfection

We adopted a siRNA-based approach to downregulate LRIG3. Summarily, three siRNA duplexes were designed to target LRIG3 and synthesized by GenePharma (Shanghai, China): siLRIG3-1 (sense (5′-3′): CCUUGAAACUUUGGACCUUTT; antisense (5′-3′): AAGGUCCAAAGUUUCAAGGTT), siLRIG3-2 (sense (5′-3′): GCUGGACCAUAACAACCUATT; antisense (5′-3′): UAGGUUGUUAUGGUCCAGCTT), siLRIG3-3 (sense (5′-3′): GGAGUAUACCACCAUCCUUTT; antisense (5′-3′): AAGGAUGGUGGUAUACUCCTT). Cells were transiently transfected with the three siRNA duplexes (each 25 pmol) using Lipofectamine RNAiMAX transfection reagent (Thermo Fisher Scientific, USA) according to the manufacturer’s instructions. After 48 h, RNA and proteins were isolated from the cells, then used to determine gene and protein expression *via* qRT-PCR and western blot assay, respectively.

### Tube Formation Assay

Matrigel (10 mg/ml; 200 µl; BD Biosciences, CA, USA) was added into wells of a 24-well plate and polymerized for 30 min at 37°C. Thereafter, HUVECs were suspended in the CM, at a density of 1×10^5^/ml, then 200 µl of the cell suspension was added to each well followed by a 12-h incubation at 37°C and 5% CO_2_. Capillary tube structures were observed, and representative images were photographed under a microscope (Carl Zeiss, Jena, Germany) at ×100 final magnification. The degree of tube formation was verified relative to the formation of tube length.

### Transwell Migration Assay

Migration assays were performed using transwell chambers according to the manufacturer’s protocol (Corning, USA). Briefly, CM was added to the lower chamber well to stimulate migration, homogeneous single-cell suspensions (2 × 10^4^/well) added to the upper chambers, then incubated for 24 h. Non-migrating cells were removed from the top well using a cotton swab, bottom cells fixed with 4% paraformaldehyde, and stained with 0.1% crystal violet. The migration rates were quantified under a microscope by counting migrated cells in five random fields per well (×100 magnification). All assays were performed in sextuplicate and repeated at least three times.

### Wound Healing Assay

Approximately 3×10^5^ HUVECs were first seeded in 6-well dishes. When the monolayer culture reached about 90% confluency, a scratch wound was marked using a 200-μl pipette tip, and all floating cells were washed off using PBS. Cells were cultured in CM at 37°C with 5% CO_2_, then wound areas captured under a microscope (Carl Zeiss, Jena, Germany) at different time points to record the wound width.

### 3-(4, 5-Dimethyl-2-thiazolyl)-2, 5-diphenyl-2-H-tetrazolium Bromide (MTT) Assay

HUVECs growth and responsiveness to CM were determined using MTT assays. Summarily, cells (2×10^3^/well) were seeded into 96-well plates and cultured with CM for 24, 48, 72, 96, and 120 h. 20 μl MTT reagent (5 mg/ml; Beyotime, Shanghai, China) was added to each well at the respective time point, and the plates incubated for 4 h at 37°C. Thereafter, the medium was replaced with 150 μl of dimethyl sulfoxide (Sigma-Aldrich, USA), and absorbance was measured at 570 nm, with 630 nm used as the reference wavelength. All assays were performed in sextuplicate and repeated at least three times.

### Western Blot Assay

Western blot assay was performed as previously described (16). Briefly, cells were first lysed in RIPA buffer, then equal amounts of protein separated on an 8 or 10% SDS-PAGE followed by electrotransfer onto a polyvinylidene difluoride membrane (Millipore, USA). The membranes were blocked, for 2 h, with 5% nonfat milk and immunoblotted with primary antibodies. After incubation with the appropriate secondary antibody, the membranes were visualized using the enhanced chemiluminescence detection system (BIO-RAD, USA). Immunoreactive bands were quantified using the densitometric analysis software (ImageJ, USA). Primary antibodies against LRIG3 (Cat #AF3495, R&D Systems), AKT (Cat #2920, Cell Signaling Technology), p-AKT (Ser473) (Cat #4060, Cell Signaling Technology), ERK (Cat #4696, Cell Signaling Technology), p-ERK (Thr202/Tyr204) (Cat #4370, Cell Signaling Technology), VEGFA (Cat #66828-1-Ig, Proteintech, Wuhan, China), and GAPDH (Cat #ab8245, Abcam) were uses for Western blot assay. GAPDH was used as an internal control.

### RNA Extraction and Quantitative Real−Time PCR (qRT-PCR)

Total RNA was extracted using the TRIzol reagent (Takara, Japan), then reverse transcribed to cDNA according to the instructions of the PrimeScript reverse transcriptase kit (Takara, Japan). qRT-PCR was performed using the TB Green™ Premix Ex Taq™ II (Takara, Japan) on an ABI 7500 real-time PCR system (Applied Biosystems, Foster City, CA, USA). Relative expression of target genes was calculated by 2^−ΔΔCt^ method, with GAPDH included as the reference gene. Experiments were performed in triplicate. All primer sequences are listed in **Supplementary Table S2**.

### Enzyme-Linked Immunosorbent Assay (ELISA)

VEGFA concentrations in CM were measured according to the manufacturer’s instructions of a commercial ELISA kit (Cat #DVE00) from R&D Systems.

### Intradermal Angiogenesis Assay

Intradermal angiogenesis assay was performed as previously described (17). Briefly, male BALB/c nude mice (4–5 weeks old; n = 6/group) were intradermally injected at the ventral skin surface with 1 × 10^6^ tumor cells in 100 μl PBS supplemented with 2% serum. The mice were sacrificed five days after tumor cell injection, then tumor-inoculated skin dissected and photographed using a digital camera. Tumor-directed capillaries were quantified by counting the number of newly formed blood vessels around the tumor-inoculated site.

### Orthotopic Xenograft Models

Orthotopic xenograft models were established as previously described (16). Summarily, cells stably expressing LRIG3 or control cells (5 × 10^5^) were first intracranially injected into male BALB/c nude mice (4–5 weeks old) (n = 6/group), then kinetics of tumor formation estimated by T2-weighted MRI after every five days. PET/CT scanning was performed to evaluate blood flow changes in orthotopic tumors, when tumor growth was angiogenesis dependent corresponding to tumor volumes of 8 to 12 mm^3^ (18, 19). Thereafter, mice were anesthetized and perfused with 4% PFA, their brains harvested and embedded in paraffin. Quantification of MVD was performed as previously described (20). Briefly, areas of highest microvascular density were examined and counted under a microscope at ×100 magnification. Results were expressed as the mean number of vessels ± standard deviations (SD) per high power field (×100). All animal experiments were conducted in accordance with the guidelines of the Institutional Animal Care and Use Committee. All animal protocols used in this study were approved by the Ethical Committee of Tongji Hospital, Tongji Medical College, Huazhong University of Science and Technology.

### Magnetic Resonance Imaging (MRI) of Orthotopic Mouse Tumors

MRI was performed on a 7 T horizontal bore Bruker small animal scanner. Summarily, mice were anesthetized using 2.5% isoflurane in 100% O_2_, and maintained with 1% to 1.5% isoflurane in 100% O_2_ delivered *via* a nose cone. Images were obtained using a T2-3D-RARE sequence (effective TE = 72 ms). Tumor sizes were determined using Amira v5.6.0 (FEI, USA).

### PET/CT Scanning and Data Acquisition

PET/CT scanning was performed as previously described (21). Briefly, mice were anesthetized with 2% isoflurane, then intravenously injected with approximately 200 ± 10 μCi ^13^N-ammonia (^13^N-NH_3_·H_2_O). After 5 min of ^13^N-NH_3_·H_2_O uptake, the mice were anesthetized with 2% isoflurane and placed on a scanning bed. PET/CT images were obtained with the static mode for 5 min, followed by CT scan on the fast mode by the TransPET Discoverist 180 system (Raycan Technology Co., Ltd, Suzhou, China). The PET images were reconstructed using the three-dimensional (3D) OSEM method with a voxel size of 0.5×0.5×0.5 mm^3^, whereas CT images were reconstructed using the FDK algorithm with 256×256×256 matrix. Images were displayed using the Pmod (Pmod Technologies LLC, Switzerland) Software, and their mean standardized uptake value (SUV) was calculated using the following formula: mean pixel value with the decay-corrected region-of-interest activity (μCi/kg)/(injected dose [μCi]/weight [kg]).

### Immunohistochemistry (IHC)

Immunohistochemical assays were performed on glioma and mouse xenograft tumor tissues as previously described (14). Expression levels of labeling were stratified and scored according to the following grading system: staining extensity was categorized as 1 (≤ 10% positive cells), 2 (< 10% and ≤ 30% positive cells), 3 (< 30 and ≤ 50% positive cells) or 4 (> 50% positive cells), whereas staining intensity was categorized as 0 (negative), 1 (weak), 2 (moderate) or 3 (strong). An overall score (0–12) was calculated for each case by multiplying the staining extensity with intensity score. Each sample was separately examined, then scored by two pathologists. In case of discrepancies in the scores, a discussion was held to reach a consensus.

### Statistical Analysis

All data, from at least three independent experiments, were expressed as means ± SD. Comparisons between groups were performed using a Student’s *t*-test. The relationship between LRIG3 levels and VEGFA expression was analyzed using a Pearson’s correlation analysis. All statistical analyses were performed using SPSS 18.0 statistical software package (IBM Corp, USA), with data followed by *p* < 0.05 considered statistically significant.

## Results

### Ectopic Expression of LRIG3 Represses Glioma Cell-Promoted Angiogenesis *In Vitro*

**Figure 1:**
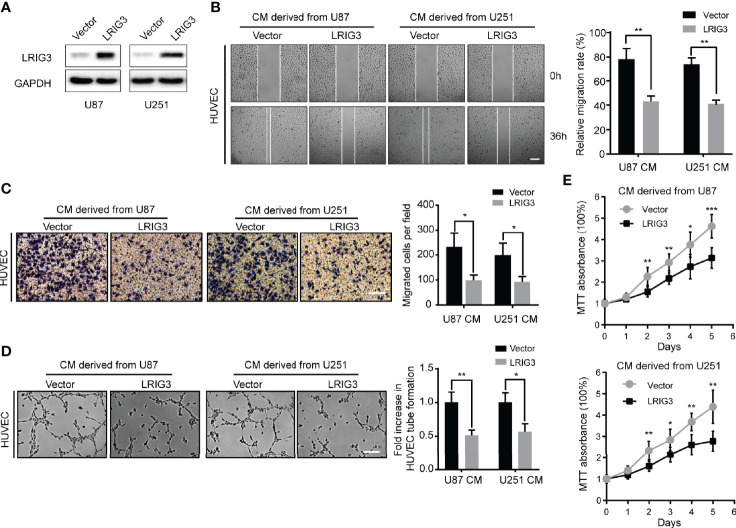
Ectopic expression of LRIG3 reduces the pro-angiogenic activity of glioma cells *in vitro.* **(A)** Western blots showing levels of LRIG3 expression in the vector control and LRIG3-transduced glioma cells. GAPDH was used as an internal control. **(B)** Representative images (left) and quantification (right) of wound healing assays in HUVECs treated with CM derived from the vector control or LRIG3-transduced glioma cells. **(C)** Representative images (left) and quantification (right) of transwell migration assays of HUVECs treated with the indicated CM. **(D)** Representative images (left) and quantification (right) of HUVECs formed tube-like structures on Matrigel-coated plates with CM derived from the vector control or LRIG3-transduced glioma cells. **(E)** HUVEC viability was determined using the MTT assay. HUVECs were treated with CM derived from the indicated cells for the specific number of days. Data are means ± SD of 3 replicates. *p < 0.05; **p < 0.01; ***p < 0.001. CM, conditioned medium.

To validate the effect of LRIG3 on angiogenesis, we performed *in vitro* gain-of-function analysis by overexpressing LRIG3 with a lentiviral vector in U87 and U251 cells. Western blots confirmed altered LRIG3 expression in glioma cells (**Figure 1A**). The wound healing assays and transwell assays revealed that ectopic expression of LRIG3 in U87 and U251 significantly suppressed the migration of HUVECs (**Figures 1B, C**). In addition, tube formation assay revealed that CM derived from LRIG3 overexpressed cells significantly inhibited HUVECs tube formation relative to CM from vector control-infected cells (**Figure 1D**). Furthermore, MTT proliferation assay revealed significantly lower cell viability of HUVEC following treatment with CM derived from LRIG3 overexpressed glioma cells (**Figure 1E**). Collectively, these results suggest that overexpressing LRIG3 suppresses glioma cell-promoted angiogenesis *in vitro*.

### LRIG3 Knockdown Enhances Pro-angiogenic Activity of Glioma Cells *In Vitro*

**Figure 2:**
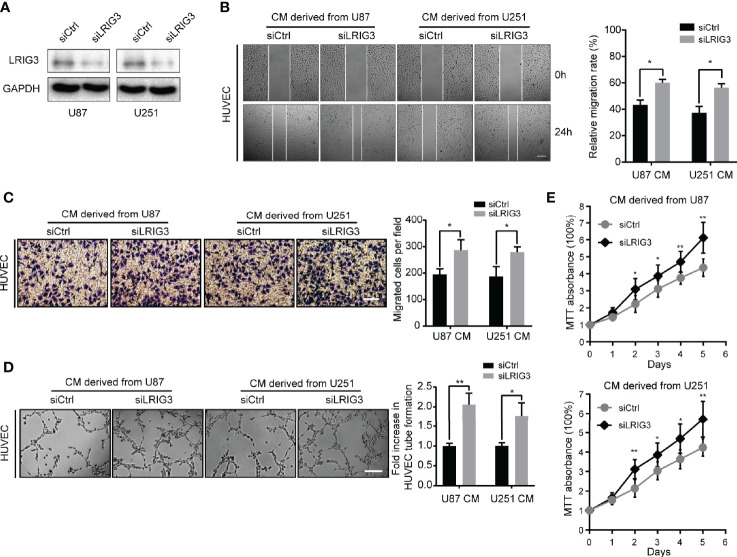
Downregulation of LRIG3 enhances the pro-angiogenic activity of glioma cells *in vitro*. **(A)** Western blot analysis of LRIG3 expression in the siRNA control and LRIG3-silenced glioma cells. GAPDH was used as an internal control. **(B)** Representative images (left) and quantification (right) of wound healing assays of HUVECs treated with CM derived from the siRNA control or LRIG3-silenced glioma cells. **(C)** Representative images (left) and quantification (right) of transwell migration assays in HUVECs treated with the indicated CM. **(D)** Representative images (left) and quantification (right) of HUVECs formed tube-like structures on Matrigel-coated plates with CM derived from the siRNA control cells or LRIG3-silenced glioma cells. **(E)** HUVEC viability was determined using the MTT assay. HUVECs were treated with CM derived from the indicated cells for the specific number of days. Data are presented as means ± SD of 3 independent replicates. *p < 0.05; **p < 0.01. CM, conditioned medium.

Since gain-of-LRIG3 could inhibit glioma cell-induced angiogenesis, we hypothesized that loss of LRIG3 could enhance the pro-angiogenic activity of glioma cells. Consequently, we transfected U87 and U251 cells with LRIG3 or control siRNAs (**Figure 2A**). Results indicated that CM derived from LRIG3 knockdown cells significantly increased the migration (**Figures 2B, C**), tube formation (**Figure 2D**), and cell viability (**Figure 2E**) of HUVECs relative to CM from siRNAs control infected cells. Taken together, these findings indicate that silencing LRIG3 enhances the pro-angiogenic activity of glioma cells *in vitro*.

### LRIG3 Inhibits the Ability of Gliomas to Induce Angiogenesis *In Vivo*

**Figure 3:**
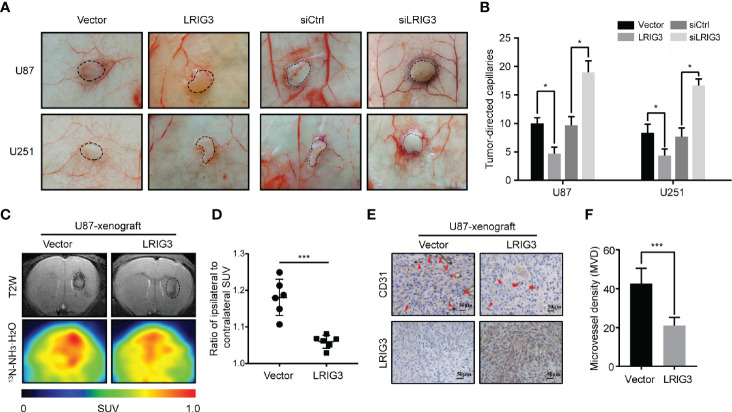
LRIG3 affects the ability of gliomas to induce angiogenesis *in vivo*. Representative images **(A)** and quantification graphs **(B)** of the intradermal glioma tumors showing tumor-directed capillaries under different conditions: U87 and U251 cells transfected with the control vector and LRIG3; U87 and U251 cells transfected with the control and LRIG3 siRNAs. Dashed circles within the images show the tumor location. **(C)** Representative MRI and ^13^NH_3_ PET scan images of intracranial xenografts bearing LRIG3-overexpressing U87 cells or control cells, 14 days after tumor cell inoculation. **(D)** SUV ratio for the tumor to reference region (T/R) was used to determine the value for angiogenesis in the tumor, n = 6/group. **(E)** IHC analysis of LRIG3 and CD31 expression in intracranial xenografts generated from LRIG3-overexpressed U87 or control cells. **(F)** Quantification of CD31+ microvessel density in intracranial xenografts generated from LRIG3-overexpressed U87 or control cells. Data are means ± SD of 3 independent replicates. *p < 0.05; ***p < 0.001. MVD, microvessel density; PET, positron emission tomography; SUV, standardized uptake value; T/R, tumor/reference.

We further investigated the role of LRIG3 in regulating the angiogenesis of glioma *in vivo* using intradermal angiogenesis assays and found that U87 cells stably overexpressing LRIG3 exhibited a significantly lower number of tumor-directed capillaries relative to cells transfected with the control vector (**Figures 3A, B**). Conversely, siLRIG3-transfected U87 cells attracted significantly more blood vessels compared to cells transfected with control siRNAs (**Figures 3A, B**). Parallel experiments performed in U251 cells revealed a similar trend. Next, we used intracranial xenograft mice to assess the effect of LRIG3 on angiogenesis and monitored tumor progression using MRI (**Figure 3C**). A ^13^N-NH_3_·H_2_O PET scan performed on day 14 after tumor cell inoculation revealed that xenografts carrying LRIG3-overexpressing U87 cells exhibited a significantly lower ^13^N-NH_3_·H_2_O uptake tumor/reference (T/R) ratio relative to the xenografts carrying control infected cells (**Figures 3C, D**). Parallel histological analysis of orthotopic xenograft tumors revealed significantly lower MVD in tumors formed by LRIG3-overexpressing U87 cells (**Figures 3E, F**). These results strongly indicate that LRIG3 overexpression suppresses glioma angiogenesis *in vivo*.

### LRIG3 Modulates VEGFA Expression *via* the PI3K/AKT Signaling Pathway

**Figure 4:**
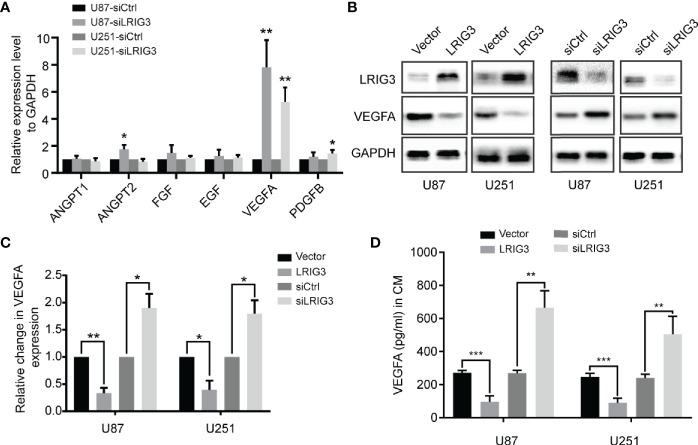
LRIG3 inhibited glioma angiogenesis by regulating VEGFA expression. **(A)** qRT-PCR analysis targeting angiogenic factors in glioma cells transduced with LRIG3 and control siRNAs. **(B)** Western blot analysis of VEGFA, LRIG3, and GAPDH. **(C)** Integrated density of the bands was normalized to GAPDH in LRIG3 knockdown or overexpression glioma cells. **(D)** Levels of VEGFA protein in the CM from glioma cells with LRIG3 knockdown or overexpression were detected by ELISA. Data are means ± SD of 3 independent replicates. *p < 0.05; **p < 0.01; ***p < 0.001. CM, conditioned medium.

To investigate the underlying mechanism, we performed qRT-PCR to quantify levels of mRNAs for angiogenesis-related factors in the glioma cells following LRIG3 knockdown or overexpression. Results showed that LRIG3 knockdown significantly elevated levels of VEGFA in U87 and U251 cells, whereas its overexpression significantly repressed these levels (**Figure 4A**, **Supplementary Figures 1A–C**). Moreover, western blots and ELISA results indicated that levels VEGFA protein were consistently upregulated and downregulated in LRIG3-knockdown and LRIG3-overexpressing glioma cells, respectively, compared to control cells (**Figures 4B–D**). These results indicate that VEGFA plays a critical effector role in LRIG3-regulated angiogenesis.

**Figure 5:**
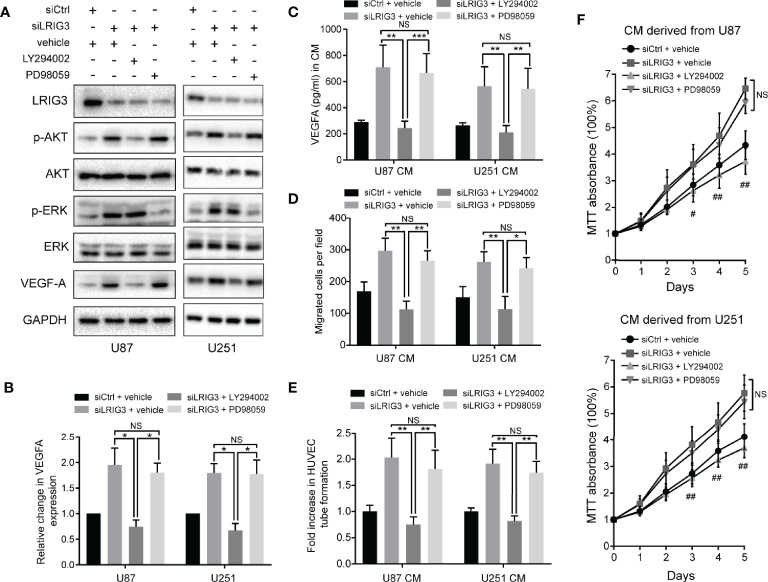
LRIG3 modulates VEGFA expression *via* the PI3K/AKT signaling pathway. Western blots showing LRIG3, p-AKT, AKT, p-ERK, ERK, and VEGFA **(A)** alongside GAPDH **(B)** in the indicated glioma cells treated with the pathway inhibitors LY294002 (25μM), PD98059 (10μM), or DMSO vehicle for 24 h. **(C)** Levels of VEGFA protein in the CM from the indicated glioma cells were detected by ELISA. **(D)** Transwell migration assays in HUVECs treated with the indicated CM. **(E)** HUVECs formed tube-like structures with the indicated CM. **(F)** Cell viability of HUVECs was determined using the MTT assay. HUVECs were treated with the CM derived from the indicated cells for the specific number of days. Data are means ± SD of 3 independent replicates. *p < 0.05; **p < 0.01; ***p < 0.001; ^#^ *p* < 0.05, siLRIG3 + LY294002 versus siLRIG3 + vehicle and siLRIG3 + PD98059; ^##^ *p* < 0.01, siLRIG3 + LY294002 versus siLRIG3 + vehicle and siLRIG3 + PD98059. CM, conditioned medium.

Our previous study has shown that LRIG3 functions as a tumor suppressor by attenuating phosphorylation of the ERK and AKT signaling pathways (13). Moreover, activation of these pathways has been associated with increased expression levels of angiogenic factors, such as ANGPT2, COX2, and VEGFA, in glioma (22). To ascertain the pathway responsible for LRIG3-mediated VEGFA expression, we used the specific inhibitors of the ERK (PD98059) and AKT (LY292004) pathways to block their activation in glioma cells following LRIG3 knockdown. As shown in **Figures 5A-C**, there was no difference in basal ERK levels in U87 and U251 when PD98059 was added. However, the increased p-ERK level upon LRIG3 silencing was reduced on PD98059 addition. Besides, the decrease in p-Akt after LY294002 treatment was more pronounced in U87 as compared to U251. Moreover, the results indicated that inhibition of the AKT, not the ERK pathway, caused changes in VEGFA expression. Correspondingly, results from functional tests of HUVECs incubated with the indicated CM showed consistent results (**Figures 5D–F**). Taken together, these results affirm that LRIG3 modulates VEGFA expression *via* the PI3K/AKT signaling pathway.

### Clinical Relevance of LRIG3, p-AKT, and VEGFA Expression in Gliomas

**Figure 6:**
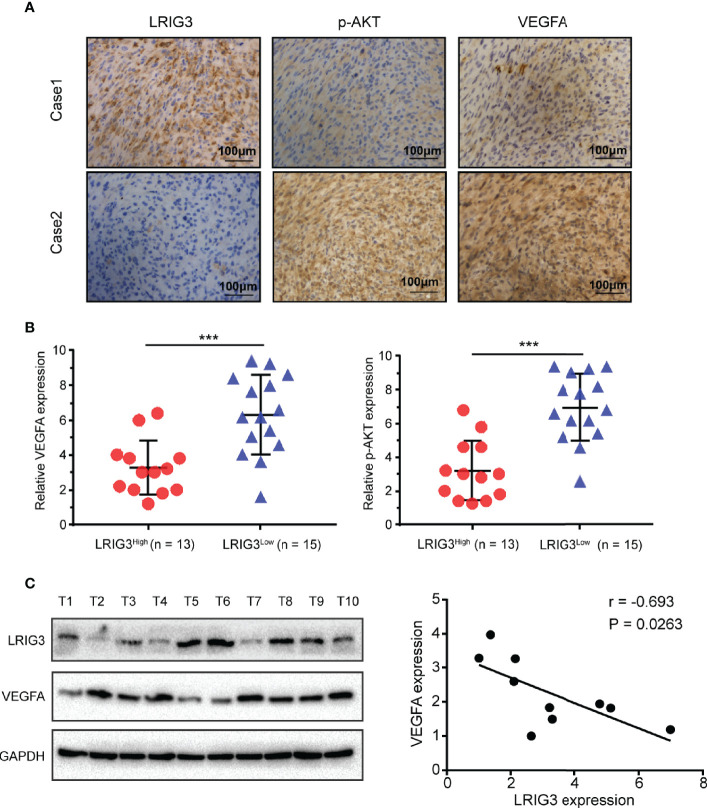
Clinical relevance of LRIG3, p-AKT, and VEGFA expression in gliomas. **(A)** IHC staining targeting LRIG3, p-AKT, and VEGFA in two representative GBM specimens. Brown staining, positive immunoreactivity. **(B)** Relative levels of LRIG3, p-AKT, and VEGFA proteins in GBM specimens (with low and high LRIG3 expression levels in 28 GBM patients). **(C)** Expression analysis (left) and correlation (right) between LRIG3 and VEGFA expression in 10 freshly collected human glioma samples. Data are means ± SD of 3 independent replicates. ***p < 0.001.

Finally, we validated the association between LRIG3 with p-AKT, and VEGFA in patients using IHC staining of these proteins in 28 glioblastoma specimens. Results revealed a significant correlation between LRIG3 and other proteins across these specimens. Specifically, tumors with high LRIG3 levels tended to express low levels of p-AKT and VEGFA, whereas the reverse was true for tumors with low LRIG3 levels (**Figures 6A, B**). Western blots corroborated the IHC results, with LRIG3 levels also strongly correlating with those of VEGFA in 10 freshly collected clinical glioma samples (**Figure 6C**). Together, these results affirmed the clinical relevance of the LRIG3/PI3K/AKT/VEGFA axis and confirmed these proteins play a vital role in the regulation of glioblastoma angiogenesis.

## Discussion

GBM, the most malignant brain tumor, is characterized by extensive neovascularization (23). Abnormal angiogenesis in glioma is considered an essential factor for metastasis and resistance of chemoradiotherapy (24, 25). Previously, we demonstrated that LRIG3 is a critical tumor suppressor in glioma (13). However, its role in glioma angiogenesis remains unclear. Herein, we identified an anti-angiogenic signature for LRIG3 and showed that its presence downregulates VEGFA both *in vitro* and *in vivo*. Stepwise investigations revealed that the PI3K/AKT pathway has a major effect on the LRIG3-mediated VEGFA expression.

The LRIG gene family was discovered in a research on EGFR, which plays a critical role in cancer progression (26). The human LRIG proteins have similar structures: a signal peptide, 15 tandem leucine-rich repeats (LRRs), three immunoglobulin-like domains, a transmembrane segment, and an intracellular domain (27). Despite their structural similarity, they may have different biological functions. LRIG1 binds to EGFR with its LRRs and immunoglobulin-like domains, then increases ligand-induced receptor ubiquitination, inhibiting the EGFR signaling pathway (28). On the contrary, LRIG2 interacts with EGFR directly and promotes activation of the downstream signaling pathway (29). Besides, LRIG3 can inhibit activation of the Ras/ERK and PI3K/AKT pathways in glioma, indicating that LRIG3, similar to LRIG1, may have an antagonistic effect against LRIG2 (13, 14). Moreover, Zeng et al. showed that LRIG3 is required for the interaction between Dual-specificity phosphatase 6 (DUSP6) and ERK in colorectal cancer. In the absence of LRIG3, DUSP6 could hardly dephosphorylate ERK, suggesting that LRIG3 has potentially more complex biological functions (10). Recently, Yang et al. reported that LRIG2 promotes glioma angiogenesis through the EGFR/VEGFA pathway (30). In this study, our findings reveal the anti-angiogenic role of LRIG3 and affirm that LRIG3 has a functionally antagonistic relationship with LRIG2 in glioma angiogenesis.

Angiogenesis is a complex process regulated by a series of molecules. Among these cytokines, VEGFA is one of the most potent pro-angiogenic molecules. It is also a critical regulator of tumor angiogenesis and is highly expressed in malignant gliomas (31). High VEGFA levels in GBM are associated with increased tumor aggressiveness and poor survival rates (32). Functionally, VEGFA primarily interacts with the VEGFR1 and VEGFR2 receptors, activating downstream signaling pathways and promoting endothelial cells migration, proliferation, and tube formation (33). VEGFA-mediated angiogenesis has been a hallmark in glioblastoma, indicating the potential value of VEGFA-targeted treatments. However, bevacizumab, a humanized antibody targeting secreted VEGFA, did not significantly improve the overall survival of patients with GBM (34). Tamura et al. suggested that the alternative pro-angiogenic mechanisms, such as vascular co-option, vasculogenesis, and vasculogenic mimicry, are induced in the development of resistance to anti-VEGFA therapy (35). Moreover, the salvage angiogenic pathways, including c-MET and STAT3, are activated in the setting of bevacizumab treatment failure (36). In this study, we screened a panel of soluble angiogenic factors involved in vascular development. We found stable up-regulation of VEGFA mRNA and protein upon depletion of LRIG3, suggesting that VEGFA is the downstream target gene for LRIG3. Furthermore, our results demonstrated their clinical relevance in glioma specimens. As mentioned above, high levels of LRIG3 is associated with the downregulation of EGFR and MET signaling in malignant gliomas, supporting the idea that LRIG3 may be a novel target for anti-angiogenic therapy.

Multiple signaling pathways, including the ERK, AKT, STAT3, and JNK pathways, have been shown to modulate VEGFA expression (37). Previously, we demonstrated that LRIG3 functions as a tumor suppressor by inactivating the ERK and AKT signaling pathways (13). To further ascertain the most important pathway, we used specific inhibitors to suppress AKT and ERK phosphorylation and found that the AKT, and not the ERK pathway, plays a major role in the LRIG3-modulated VEGFA expression. Activation of the PI3K/AKT pathway in glioma cells can increase VEGFA secretion, both *via* hypoxia-inducible factor 1 (HIF-1) dependent and independent mechanisms (25, 38). Additionally, the PI3K/AKT pathway’s hyperactivation affects numerous biological processes in glioma, including angiogenesis, cytoskeletal rearrangement, cell proliferation, and vasculogenic mimicry formation (39, 40). Apart from these, VEGFA binding to VEGFR1 enhances a variety of signaling pathways, including the ERK and AKT pathways, leading to tumor invasion and migration (41). Whether LRIG3 has an inhibitory effect on this positive feedback loop warrants further investigation. Based on the results above, it is evident that LRIG3 represents a promising therapeutic target for developing anti-angiogenic strategies against gliomas.

Our study had several limitations. On the one hand, we only analyzed a series of classical angiogenesis-related factors, limiting the study’s comprehensiveness. On the other, this was a single-center study with a small sample size. Multicenter data using larger clinical sample sizes are needed to validate our results. Further investigations are still required to identify the specific interactions between LRIG3 protein and the PI3K/AKT signaling pathway, as well as other relevant signaling pathways that might play potential roles in glioma angiogenesis.

In summary, we have demonstrated that LRIG3 inhibits angiogenesis in glioma. Specifically, our results show that LRIG3 suppresses expression and secretion of VEGFA, both *in vitro* and *in vivo*, by inactivating the PI3K/AKT signaling pathway. This novel LRIG3/PI3K/AKT/VEGFA axis provides new insights into the underlying mechanisms of glioma angiogenesis. This axis may be a potential target for developing therapeutic approaches for treating patients with malignant gliomas.

## Data Availability Statement

The original contributions presented in the study are included in the article/**Supplementary Material**. Further inquiries can be directed to the corresponding author.

## Ethics Statement

The studies involving human participants were reviewed and approved by the Research Ethics Committee of Tongji Hospital, Tongji Medical College, Huazhong University of Science and Technology. The patients/participants provided their written informed consent to participate in this study. The animal study was reviewed and approved by Ethical Committee of Tongji Hospital, Tongji Medical College, Huazhong University of Science and Technology.

## Author Contributions

CP and DG designed the study. CP, HC, and YL collected cancer samples. CP, HY, PQ, and BM performed the experiments. CP analyzed data and drafted the manuscript. BW, QD, and FM revised the manuscript. All authors contributed to the article and approved the submitted version.

## Funding

This project was supported by the National Natural Science Foundation of China (grant no. 81874086) and the Natural Science Foundation of Hubei Province, China (grant no. 2018CFB579).

## Conflict of Interest

The authors declare that the research was conducted in the absence of any commercial or financial relationships that could be construed as a potential conflict of interest.
