# Supplementary figures and images for: LRIG3 Suppresses Angiogenesis by Regulating the PI3K/AKT/VEGFA Signaling Pathway in Glioma
Source: Front Oncol. 2021 Feb 25;11:621154. doi: 10.3389/fonc.2021.621154 (PMC7946980; doi:10.3389/fonc.2021.621154)

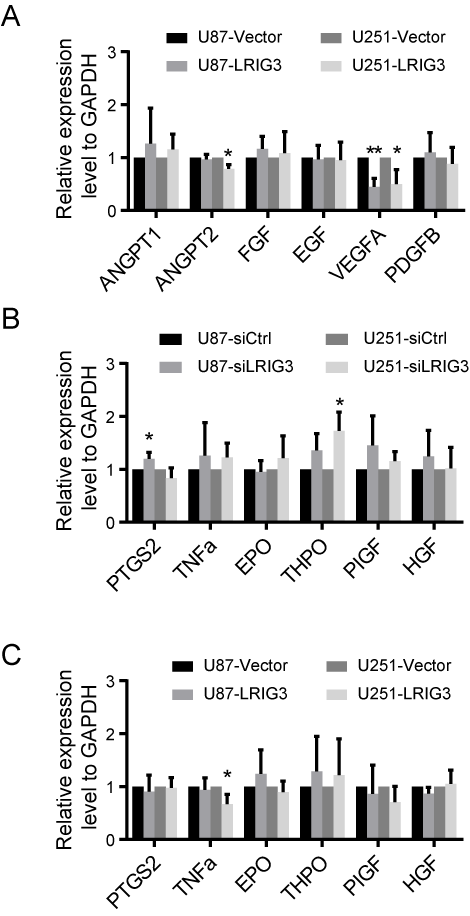

Supplement: Supplementary file 1 [file Image_1.tif]
