# Supplementary material for: LRIG3 Suppresses Angiogenesis by Regulating the PI3K/AKT/VEGFA Signaling Pathway in Glioma
Source: Front Oncol. 2021 Feb 25;11:621154. doi: 10.3389/fonc.2021.621154 (PMC7946980; doi:10.3389/fonc.2021.621154)
Supplement: Supplementary file 2 [file Table_1.docx]

Table S1. Characteristics of 28 GBM patients

| Characteristic | | Value |
| --- | --- | --- |
| Total sample (n) | | 28 |
| Sex (n) | |  |
|  | Male | 16 |
|  | Female | 12 |
| Medium age, years (range) | | 56（30-72） |
| Tumor location | |  |
|  | Frontal | 13 |
|  | Non-frontal | 15 |

These samples were collected from September 2018 to December 2019.

Abbreviations: GBM, glioblastoma
