# Supplementary material for: LRIG3 Suppresses Angiogenesis by Regulating the PI3K/AKT/VEGFA Signaling Pathway in Glioma
Source: Front Oncol. 2021 Feb 25;11:621154. doi: 10.3389/fonc.2021.621154 (PMC7946980; doi:10.3389/fonc.2021.621154)
Supplement: Supplementary file 3 [file Table_2.docx]

Table S2. Primers

| Primers used for real-time RT-PCR | |
| --- | --- |
| GAPDH-Forward | GGAGCGAGATCCCTCCAAAAT |
| GAPDH-Reverse | GGCTGTTGTCATACTTCTCATGG |
| LRIG3-Forward | ATCTCAGCTATCCCACCCAAG |
| LRIG3-Reverse | AAGCCGTAAAGCCAGCCTT |
| ANGPT1-Forward | AGCGCCGAAGTCCAGAAAAC |
| ANGPT1-Reverse | TACTCTCACGACAGTTGCCAT |
| ANGPT2-Forward | AACTTTCGGAAGAGCATGGAC |
| ANGPT2-Reverse | CGAGTCATCGTATTCGAGCGG |
| EGF-Forward | TGGATGTGCTTGATAAGCGG |
| EGF-Reverse | ACCATGTCCTTTCCAGTGTGT |
| FGF-Forward | TCCTGCCAACTTTGCTCTACA |
| FGF-Reverse | CAGGGCTGGAACAGTTCACAT |
| PDGFB-Forward | CTCGATCCGCTCCTTTGATGA |
| PDGFB-Reverse | CGTTGGTGCGGTCTATGAG |
| VEGFA-Forward | AGGGCAGAATCATCACGAAGT |
| VEGFA-Reverse | AGGGTCTCGATTGGATGGCA |
| EPO-Forward | GGAGGCCGAGAATATCACGAC |
| EPO-Reverse | CCCTGCCAGACTTCTACGG |
| TNFa-Forward | CCTCTCTCTAATCAGCCCTCTG |
| TNFa-Reverse | GAGGACCTGGGAGTAGATGAG |
| PTGS2-Forward | CTGGCGCTCAGCCATACAG |
| PTGS2-Reverse | CGCACTTATACTGGTCAAATCCC |
| PIGF-Forward | TGACATGGTTGTGCATCTGTT |
| PIGF-Reverse | ACTCTATCAGTGGTGCTCCATAC |
| HGF-Forward | GCTATCGGGGTAAAGACCTACA |
| HGF-Reverse | CGTAGCGTACCTCTGGATTGC |
| THPO-Forward | AACTGCAAGGCTAACGCTGT |
| THPO-Reverse | GACATGGGAGTCACGAAGCA |
